# FeatSNP: An Interactive Database for Brain-Specific Epigenetic Annotation of Human SNPs

**DOI:** 10.3389/fgene.2019.00262

**Published:** 2019-04-02

**Authors:** Chun-yu Ma, Pamela Madden, Paul Gontarz, Ting Wang, Bo Zhang

**Affiliations:** ^1^Center of Regenerative Medicine, Department of Developmental Biology, Washington University School of Medicine, St. Louis, MO, United States; ^2^Department of Psychiatry, Washington University School of Medicine, St. Louis, MO, United States; ^3^Department of Genetics, The Edison Family Center for Genome Sciences & Systems Biology, Washington University School of Medicine, St. Louis, MO, United States

**Keywords:** SNP, database, epigenetics, brain, transcription factor

## Abstract

FeatSNP is an online tool and a curated database for exploring 81 million common SNPs’ potential functional impact on the human brain. FeatSNP uses the brain transcriptomes of the human population to improve functional annotation of human SNPs by integrating transcription factor binding prediction, public eQTL information, and brain specific epigenetic landscape, as well as information of Topologically Associating Domains (TADs). FeatSNP supports both single and batched SNP searching, and its interactive user interface enables users to explore the functional annotations and generate publication-quality visualization results. FeatSNP is freely available on the internet at FeatSNP.org with all major web browsers supported.

## Introduction

Genome-wide association studies (GWAS) and expression quantitative trait loci (eQTL) analyses have identified thousands of genetic variants that are associated with a wide range of human phenotypes, shedding lights on the understanding of the genetic effect to human diseases. However, a key challenge for scientists in the human genetics community is to understand the molecular mechanism connecting significant genetic variant and specific phenotype. More than 90% of SNPs associated with human phenotypes are located in non-protein-coding regions, and cannot be explained by alteration of amino acid sequence of proteins (Welter et al., 2014). Recently, mounting evidence suggests that disease-associated non-coding SNPs are highly enriched in tissue-specific regulatory elements including enhancers, which can be detected and defined by specific chromatin modifications (Carey et al., 2015; Zhou et al., 2015; Agrawal et al., 2018). Moreover, some non-coding SNPs are found to be located within transcription factor (TF) binding motifs, which affect the TF binding affinity and result in allele switching and/or allele-specific regulation of target genes (Andersson et al., 2014; Roadmap Epigenomics et al., 2015; Nelson et al., 2016). These evidences underscore the potential causal role of non-coding genetic variants in affecting human diseases and phenotypes through regulation of gene expression (Claussnitzer et al., 2015).

Here we introduce FeatSNP, an online tool and database which provides an interactive user interface (UI) for inquiring brain-specific functional and epigenetic annotation of human SNPs. Unlike traditional SNP functional annotation databases, such as RegulomeDB (Boyle et al., 2012) and HaploReg (Ward and Kellis, 2012), FeatSNP focuses on the collection and curation of brain-specific functional genomics data, including epigenomes, transcriptomes, and eQTL data, to better annotate the regulatory potential of single SNP. Specifically, FeatSNP supplies a series of new features to facilitate research understanding the functional annotation of SNP on human brain (Supplementary Table S1). FeatSNP uses human brain transcriptomes to improve and refine the prediction of allele-specific TF binding motifs. The expression correlation between SNP-associated gene and predicted SNP-associated TFs was used to determine the best allele-associated TF candidate. The interactive UI allows the users easily to browse functional annotation and generate analysis results and high quality figures.

## Methods

**FIGURE 1:**
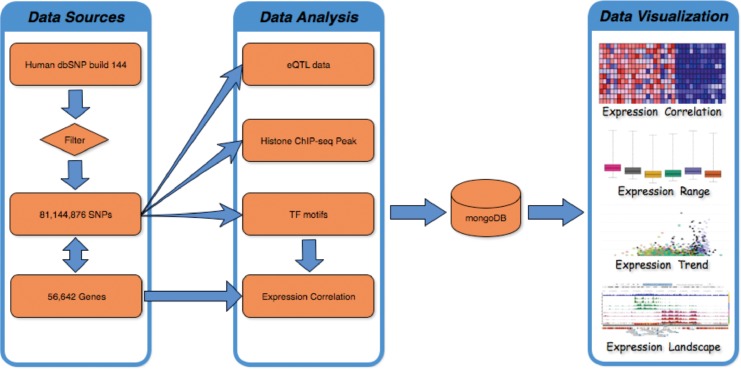
Analysis flowchart and infrastructure of FeatSNP.

FeatSNP consists of a front end UI implemented with HTML/PHP/JavaScript, and a backend NoSQL database implemented with MongoDB (v3.2.7) as shown in Figure 1. The current SNP dataset contains 81,144,876 bi-allelic SNPs from dbSNP (V144), with SNP accession number as unique identifier in the database. Human dbSNP build 144 was downloaded from ftp.ncbi.nih.gov/snp, which includes 84,435,229 SNPs records, 1,591,294 insertions records, 2,595,517 deletions records, 33,234 indel records, and 110 Multiple Nucleotide Polymorphisms (MNPs) records. After filtering redundant records, 81,144,876 of 84,435,229 biallelic SNPs were used to generate functional annotations and were curated by the FeatSNP database. The genome coordinates (hg19) of 81,144,876 SNPs were used to associate the SNPs with their nearest genes based on 56,642 records of GENOCDE gene annotation Release 19 (GRCh37.p13).

To predict impact of allele-specific TF binding affinity by SNPs, the Position Weight Matrix (PWM) of 519 vertebrate TFs were collected from JASPAR (Core Vertebrate 2016) (Mathelier et al., 2016). After evaluating the motif weight PWM of 519 TFs at base-pair resolution (Supplementary Figure S2), the reference and alternate alleles for every SNP with flanking 10 bp of genomic sequences both upstream and downstream were obtained from the UCSC Genome Browser. FIMO (Grant et al., 2011) was used to scan the 21 bp sequence to identify binding motifs matching any of the 519 TF PWMs, and calculate the TFBS motif scores. Only instances where a motif in the sequence (i) passed the threshold of *P* < 1e-2 in either the reference or the alternate allele, and (ii) contained the SNP location and (iii) the difference of motif scores between the reference and the alternate allele was greater than 2, were recorded in the database.

1,259 transcriptome datasets of 13 brain tissues generated by the GTEx consortium (Gibson, 2015) were used to calculate the Pearson correlation between each SNP associated gene and predicted binding TFs. The lowly expressed gene and TFs (expression of all samples in one tissue less than 0.2RPKM) were removed. The correlation and gene expression in 13 brain tissues were visualized by using JavaScript package Highcharts (v5.0.2).eQTL data of 10 brain tissues generated by GTEx consortium were negative-log10 transformed and further visualized by using Highcharts (v5.0.2).

Histone modification ChIP-seq data of 10 brain tissues were downloaded from NIH Roadmap Epigenomics data portal. Bedtools was used to identify SNPs residing in peaks of 7 histone modification marks (H3K4me3, H3K36me3, H3K27me3, H3K4me1, H3K27ac, H3K9me3, and H3K9ac) that were identified by macs2 (Zhang et al., 2008) with default parameters. To enhance the user experience, the WashU epigenome browser (Zhou et al., 2015) was embedded in the UI to display epigenetic landscape in a 200 bp region surrounding each SNP. The browser also displays DNA methylation data (Whole Genome Bisulfite Sequencing) of 4 neuronal progenitor and brain tissues generated by Roadmap Epigenomics Project, enhanced epilogos visualization ^1^ of all 127 epigenomes, and topologically associating domains (TAD) data of GM12878, IMR90, and Hap1 cell lines (Rao et al., 2014; Sanborn et al., 2015). eQTL data of 10 brain tissues generated by GTEx consortium were also visualized on the embedded WashU epigenome browser.

The association records of SNP and human disease/traits (V1.0.2) were downloaded from GWAS Catalog. 33,894 associations with *p*-value smaller then 5E-8 were kept and classified based on 1,374 human disease/traits categories. The functional annotations of these 33,894 SNPs were reported on FeatSNP.org/html_file/disease_classification.html (Supplementary Figure S3).

## Results

**FIGURE 2:**
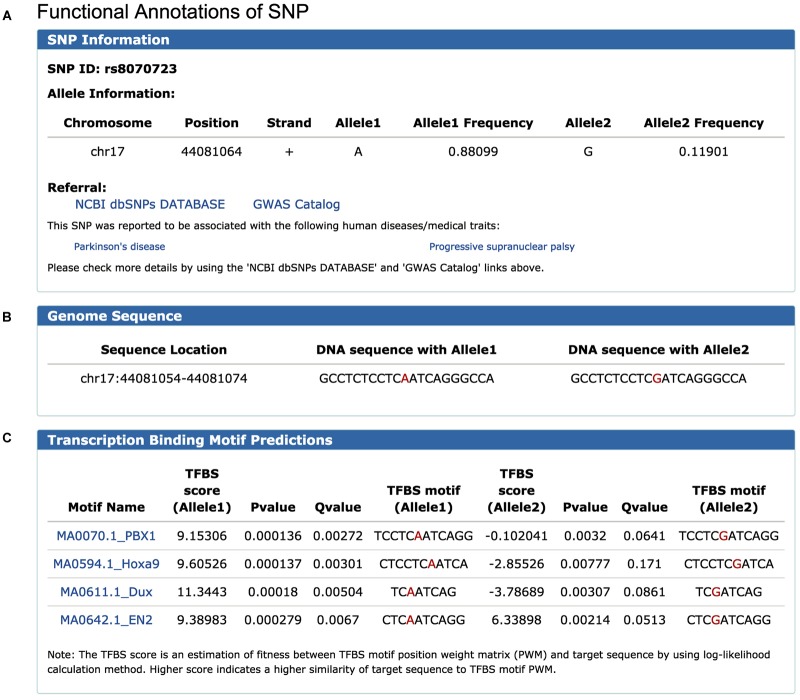
Information of SNP rs8070723 retrieved from FeatSNP. **(A)** Genomic information of rs8070723, with external links to NCBI dbSNPs and GWAS Catalog. **(B)** DNA sequence around rs8070723. **(C)** Predicted TFs binding motifs containing rs8070723 with A/G alleles.

To illustrate the use of FeatSNP, we performed the analysis using rs8070723 as an example. rs8070723 is an intronic A/G SNP (major allele A frequency 0.881, minor allele G frequency 0.119) in *MAPT*, the gene that encodes the microtubule-associated protein tau, and is associated with Progressive Supranuclear Palsy (Hoglinger et al., 2011) and with Parkinson’s Disease (UK Parkinson’s Disease Consortium et al., 2011). To better understand the regulatory potential of this human disease-associated SNP, we inquired the epigenetic annotation of rs8070723 in FeatSNP through Single SNP ID Searching function on SNP Query Page (Supplementary Figure S4). The database first reported the basic information of SNP rs8070723, including genomic location, allelic frequency, surrounding DNA sequence, and associated gene (Figure 2A–C). Users can further access the genetic information and associated human disease or traits of inquired SNPs on dbSNP and GWAS Catalog through external links.

FeatSNP found four potential TF binding motifs harboring rs8070723 with A allele, including *PBX1*, *Hoxa9*, *Dux*, and *EN2*. All four TF binding motifs had high TFBS scores in A allele, and the TFBS motifs were destroyed with G allele with low TFBS scores (Figure 2C). *PBX1* encodes a nuclear protein that belongs to the *PBX* homeobox family of transcriptional factors, and studies suggested *PBX1* regulates the patterning of the cerebral cortex (Golonzhka et al., 2015) and its transcriptional network controls dopaminergic neuron development in Parkinson’s disease (Villaescusa et al., 2016). *EN2* encodes homeodomain-containing proteins and has been implicated in the control of pattern formation during development of the central nervous system (Genestine et al., 2015). *Hoxa9* is an important homeobox transcription factor and plays important roles in myeloid leukemogenesis (Siriboonpiputtana et al., 2017). Dux-family transcription factors were recently identified to regulate zygotic genome activation in placental mammals (De Iaco et al., 2017). Thus, *PBX1* and *EN2* could be the potential master TFs affected by the SNP rs8070723.

**FIGURE 3:**
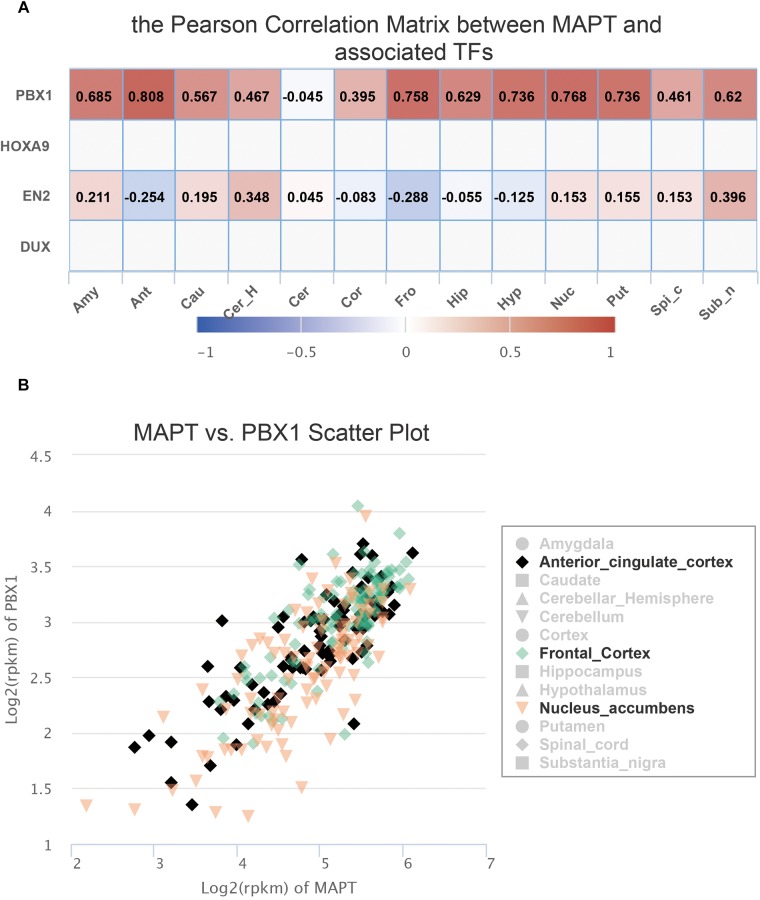
Expression information of SNP rs8070723 tagged gene retrieved from FeatSNP. **(A)**. Pearson Correlation Matrix between rs8070723 tagged MAPT and potential bound TFs. **(B)**. Scatter plot of gene expression of MAPT and PBX1 in anterior cingulate cortex, frontal cortex, and nucleus accumbens.

Since FeatSNP curated 1,259 transcriptome data of 13 brain tissues generated by the GTEx consortium (Gibson, 2015), we were able to further check the expression level of *PBX1* and *EN2* in multiple brain regions in FeatSNP database. *EN2* was only expressed in the cerebellum of the brain (Supplementary Figure S1A) and did not correlate with expression level of *MAPT* (Figure 3A). We found that PBX1 highly expressed in different brain regions (Supplementary Figure S1B), and we also found the expression of *MAPT* had strong and specific correlation with *PBX1* in multiple brain regions (Figure 3A), especially in anterior cingulate cortex (*r* = 0.808), nucleus accumbens (*r* = 0.768), and frontal cortex (*r* = 0.768) (Figure 3B), which were considered as major affected regions of Progressive Supranuclear Palsy (Salmon et al., 1997).

**FIGURE 4:**
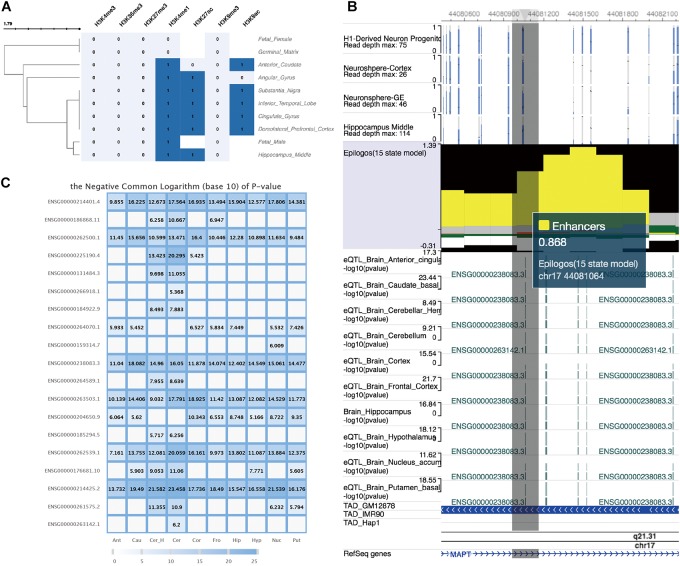
Epigenetic annotation and eQTL information of SNP rs8070723 tagged genomic loci retrieved from FeatSNP. **(A)** Clustering visualization of epigenetic annotation to the genomic loci tagged by SNP rs8070723. **(B)** WashU EpiGenome Brower view of genomic loci tagged by rs8070723. Top: DNA methylation level of CpG sites in four neuronal cells. Middle track: Epilogos visualization of chromHMM predicted chromatin status. Followed eQTL tracks: log10 transformed *p*-value of eQTL in 10 brain regions. TAD track: Topological Associated Domain tracks of GM12878, IMR90, and Hap1. Bottom: RefSeq gene annotation track. **(C)** Complete eQTL information of SNP rs8070723 in 10 brain regions.

We further explored the epigenetic annotation of the genomic regions tagged by rs8070723 in 10 brain regions by using epigenome data generated from Roadmap Consortium, which were also curated in FeatSNP database. We found the regions tagged by SNP rs8070723 enriched for strong active histone modification signals including H3K4me1, H3K9ac, and H3K27ac in 8 brain tissues (Figure 4A). Such active histone modifications were generally associated with active enhancer and promoter functions. Chromatin epigenetic status prediction based on chromHMM (Ernst and Kellis, 2012) suggested that the regions tagged by SNP rs8070723 could be considered as strong enhancers (Figure 4B). Finally, we explored the eQTL data in 13 brain tissues, and found rs8070723 was associated with several genes’ expression, including *MAPT* (Figure 4B,C). *MAPT* gene mutations have been associated with several neurodegenerative disorders such as Alzheimer’s disease and Parkinson’s disease. Our result suggests that rs8070723 G allele might influence *MAPT* expression level by reducing the binding affinity of upstream regulatory protein *PBX1*, therefore providing a mechanistic association with neurodegenerative diseases including Progressive Supranuclear Palsy and Parkinson’s Disease.

## Conclusion

In summary, FeatSNP is an interactive database providing brain-specific functional genomics resources to investigate the regulatory potential of human SNPs. This database provides a multitude types of functional annotations, including TF binding motif prediction, epigenetic landscape, expression correlation and eQTL information. We anticipate that this database will facilitate scientists to investigate the functional impact of their candidate genetic variants in a more streamlined, rapid, and efficient fashion.

## Data Availability

Publicly available datasets were analyzed in this study. This data can be found here: http://www.roadmapepigenomics.org/.

## Author Contributions

C-yM and BZ performed the data analysis, C-yM and PG developed the database and website. PM, TW, and BZ designed and supervised the study.

## Conflict of Interest Statement

The authors declare that the research was conducted in the absence of any commercial or financial relationships that could be construed as a potential conflict of interest.

## Footnotes

1 epilogos.altiusinstitute.org
