# Supplementary material for: FeatSNP: An Interactive Database for Brain-Specific Epigenetic Annotation of Human SNPs
Source: Front Genet. 2019 Apr 2;10:262. doi: 10.3389/fgene.2019.00262 (PMC6454007; doi:10.3389/fgene.2019.00262)

**Supplementary Table 1. Comparison between FeatSNP and other genetic variants annotation tools and databases**

|  | **FeatSNP** | GenomeRunner | Genomic Association Tester | LOLA (Locus Overlap Analysis) | EpiMine | Genomic Hyperbrowser | RegulomeDB | HaploReg |
| --- | --- | --- | --- | --- | --- | --- | --- | --- |
| Summary | FeatSNP is an online tool and a curated database for exploring common SNPs' potential functional impact on the human brain. | Functional enrichment analysis of SNP sets within regulatory/ epigenomic context | Fests for association between intervals in a genome analysis context | LOLA is an R Bioconductor package for genomic locus overlap enrichment. | EpiMINE performs genome-wide quantitative and correlative analyses. | The GSuite HyperBrowser system includes a range of tools to handle acquisition, processing and analysis of genomic tracks. | RegulomeDB identifies DNA features and regulatory elements in non-coding regions of the human genome. | HaploReg is a tool for exploring annotations of the noncoding genome at variants on haplotype blocks. |
| Infrastructure | Web | Web | Python | R | Python | Web | Web | Web |
| User interface | Yes | Yes | No | No | Yes | Yes | Yes | Yes |
| Epigenetic annotation | Roadmap | Cistrome, ENCODE, Roadmap, Fantom | Not specified | Cistrome, ENCODE, Roadmap | Not specified | ENCODE, Roadmap | ENCODE | ENCODE, Roadmap |
| Allelic-associated TF binding prediction | Yes | No | No | No | No | No | No | Yes |
| Sequence conservation | Yes | No | No | No | No | Yes | No | No |
| eQTL information | Yes | No | No | No | No | No | No | Yes |
| Transcriptome information | Yes | No | Not specified | Not specified | Not specified | Not specified | No | No |
| TADs information | Yes | No | No | No | No | Not specified | No | Yes |
| Real-time genome browser operation | Yes, embedded | No | No | No | No | Not specified | Link out | No |
| Publication quality visualization | Yes | Yes | Yes | Yes | Yes | No | No | No |

**Supplementary Figure 1. Expression of EN2 and PBX1 in 13 brain regions**

**Supplementary Figure 2. Summary of TFBS PWMs and maximized weight at base-pair resolution in 21bp window.**

Figure legend. A). Percentage of length of 519 TFBS motif. B). Distribution of max weight at each nucleotide position of 519 TFBS motifs in 21bp window. C). 21bp motif logo of IRF1 and MAFG.

**Supplementary Figure 3. 1,374 know human disease or medical traits recorded in GWAS Catalog.**


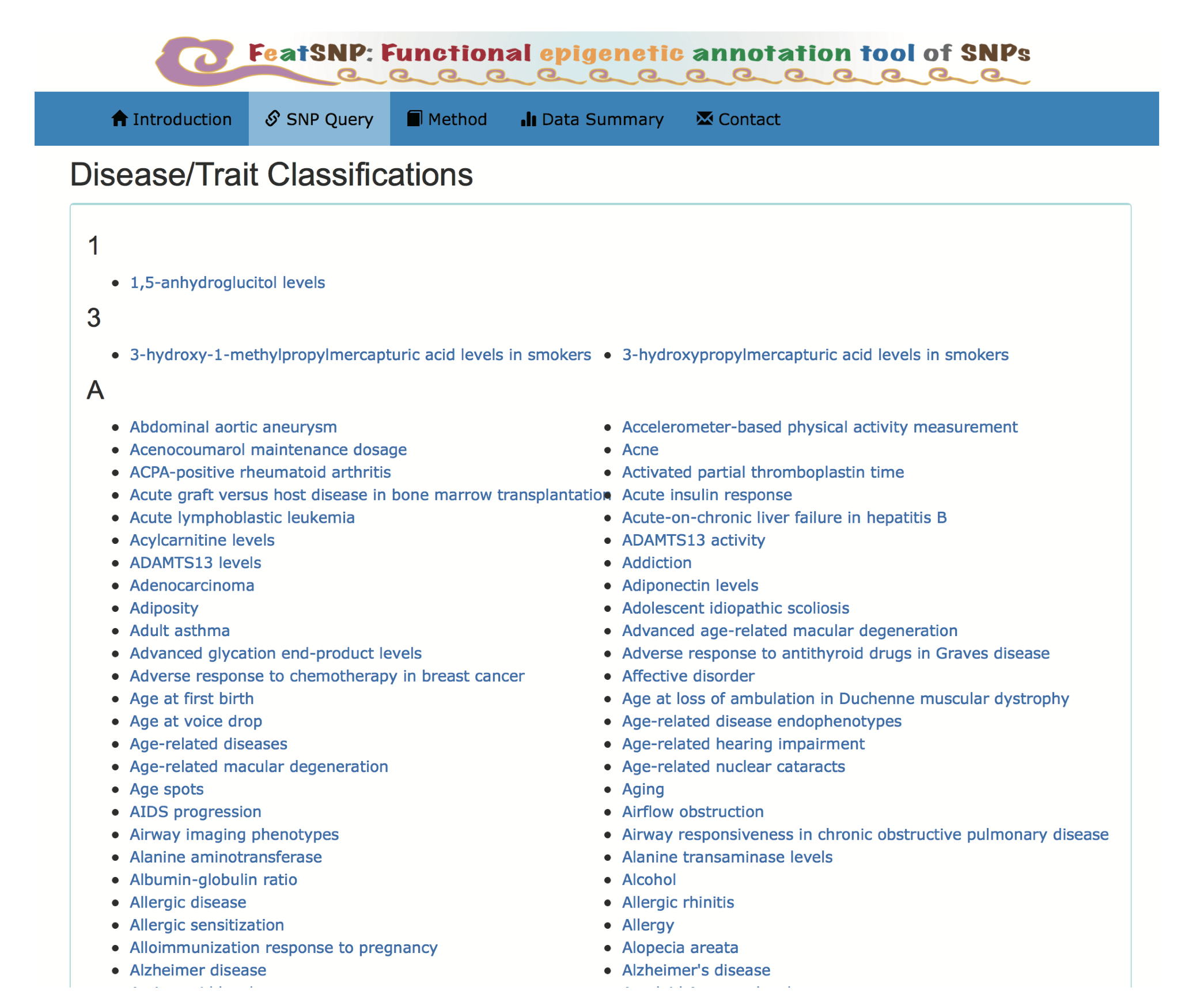


**Supplementary Figure 4. SNP Query page.**


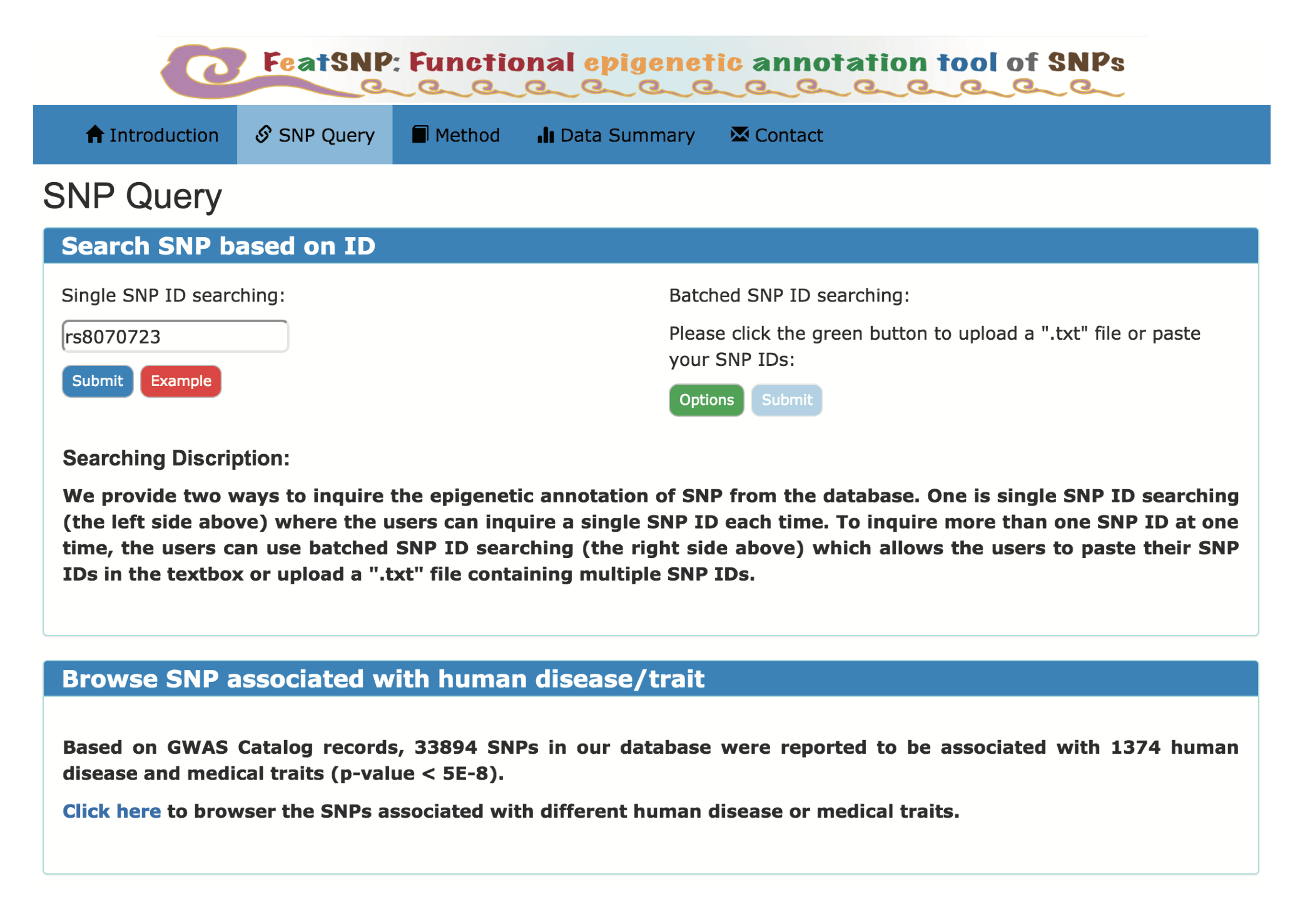

Supplement: Supplementary file 1 [file Data_Sheet_1.docx]
